# Leisure Time Physical Inactivity and Sedentary Behaviour and Lifestyle Correlates among Students Aged 13–15 in the Association of Southeast Asian Nations (ASEAN) Member States, 2007–2013

**DOI:** 10.3390/ijerph13020217

**Published:** 2016-02-15

**Authors:** Karl Peltzer, Supa Pengpid

**Affiliations:** 1 ASEAN Institute for Health Development, Mahidol University, Salaya, Phutthamothon, Nkohn Pathom 73170, Thailand; supaprom@yahoo.com; 2 Department of Research & Innovation, University of Limpopo, Turfloop Campus, Sovenga 0727, South Africa; 3 HIV/AIDS/STIs and TB (HAST), Human Sciences Research Council, Pretoria 0001, South Africa

**Keywords:** physical inactivity, sedentary behaviour, prevalence, overweight, health risk behaviour, mental health, protective factors, ASEAN

## Abstract

The aim of this study was to examine the relationship between self-reported leisure time physical inactivity frequency and sedentary behaviour and lifestyle correlates among school children in the Association of Southeast Asian Nations (ASEAN) region. The analysis included 30,284 school children aged 13–15 years from seven ASEAN countries that participated in the Global School-based Student Health Survey (GSHS) between 2007 and 2013. The measure asked about overall physical activity, walking or biking to school, and on time spent sitting. Overall, the prevalence of physical inactivity was 80.4%, ranging from 74.8% in Myanmar to 90.7% in Cambodia and sedentary behaviour 33.0%, ranging from 10.5% in Cambodia and Myanmar to 42.7% in Malaysia. In multivariate logistic regression, not walking or biking to school, not attending physical education classes, inadequate vegetable consumption and lack of protective factors (peer and parental or guardian support) were associated with physical inactivity, and older age (14 and 15 years old), coming from an upper middle income country, being overweight or obese, attending physical education classes, alcohol use, loneliness, peer support and lack of parental or guardian supervision were associated with sedentary behaviour. In boys, lower socioeconomic status (in the form of having experienced hunger) and coming from a low income or lower middle income country were additionally associated with physical inactivity, and in girls, higher socioeconomic status, not walking or biking to school and being bullied were additionally associated with sedentary behaviour. In conclusion, a very high prevalence of leisure physical inactivity and sedentary behaviour among school going adolescents in ASEAN was found and several factors identified that may inform physical activity promotion programmes in school-going adolescents in ASEAN.

## 1. Introduction

According to the World Health Organization [1],”In order to improve cardiorespiratory and muscular fitness, bone health, and cardiovascular and metabolic health biomarkers: Children and youth aged 5–17 should accumulate at least 60 min of moderate- to vigorous-intensity physical activity daily.” According to Hancock, Inchley and HBSC’s Physical Activity Focus Group [2], “Most state that adolescents should spend no more than 2 h per day engaging in sedentary screen-based activities such as TV viewing.” “In particular, the evidence suggests that daily TV viewing in excess of 2 h is associated with reduced physical and psychosocial health.” [3]. Several guidelines and studies define physical inactivity as obtaining less than 60 min of moderate to vigorous-intensity physical activity per day on at least 5 days per week and “sedentary” behaviour as spending 3 or more hours per day sitting (excluding in school or doing homework) [2,4,5,6,7,8]. Physical inactivity and sedentary behaviour have been shown to be separate constructs, independently contributing to adverse health outcomes [9,10]. The period of transition from childhood to adolescence has been shown to be associated with a significant decline in physical activity [11,12], while the adolescence period is an ideal time for the adoption of physical activity behaviours [11].

Guthold *et al*. [4] found among school-going adolescents (13–15 years) in 34 mainly low and middle income countries participating in the Global School-based Student Health Survey (GSHS) between 2003 and 2007 that overall, 76.2% of boys and 84.6% of girls were physically inactive (not meeting recommendations of 5 or more days/week of at least 60 min of moderate or vigorous-intensity physical activity), in eight African countries 85.8% were physically inactive [5], in ten Eastern Mediterranean countries the prevalence of physical inactivity was 81% [6] and among school going children aged 10 to 17 years in Malaysia 57.3% were physically inactive [13]. Further, in the 34 country study of the GSHS in more than half of the countries more than a third of the students engaged in sedentary behaviour [4], while in a more recent study in 10 Eastern Mediterranean countries Al Shuhi *et al*. [6] found an overall prevalence of sedentary behaviour of 29%. There is a lack of national studies on physical inactivity and sedentary behaviour in adolescents in the Association of Southeast Asian Nations (ASEAN) region (Brunei, Cambodia, Indonesia, Laos, Malaysia, Myanmar, Philippines, Singapore, Thailand, and Vietnam) [14,15].

Several socio-demographic indicators have been found to be associated with physical inactivity in adolescents, such as being female [6,13,16,17], younger age group [17], older age [13], higher socioeconomic status [16] and low social class [18,19], and associations with sedentary behaviour in adolescents included being female [6,16] and higher socioeconomic status [16].

Regarding health risk status or excess body mass index (BMI), obesity was related to physical inactivity in several studies, but not in other studies [13,17,20], while in general, having a higher BMI was positively associated with sedentary behaviours [3,20,21]. In terms of health risk behaviour, negative health indices/health risk behaviour [22] such as substance use [23], bullying [19], health complaints [23], passive transportation [17], sedentary behaviour [17], not attending physical education classes [24,25], and inadequate fruit and vegetable consumption [26] were found to be associated with physical inactivity and negative health indices/health risk behaviour such as substance use [5,23,27,28], skipping breakfast [13], health complaints [23] and attending physical education classes [25] were associated with sedentary behaviour.

Concerning mental health indicators and physical inactivity, physical inactivity was associated “with reports of feeling nervous among girls, and with feeling low, irritability, and feeling nervous among boys” [29], and in Central Europe with psychological complaints [30]. Suchert *et al*. [31] found in a systematic review on sedentary behavior and indicators of mental health in adolescents that “high levels of screen time were associated with more hyperactivity/inattention problems and internalizing problems as well as with less psychological well-being and perceived quality of life”, and Liu *et al*. [32] found in a meta-analysis that screen time in children and adolescents was correlated with depression risk in a non-linear dose-response manner.

In terms of protective factors, Van Der Horst *et al*. [33] identified in a review of adolescents attending physical education classes positive associations between parental attitude, family influences, friend support and physical activity. Little information is available about the relationship between physical inactivity, sedentary behaviour and health risk status and behaviour, mental health and protective factors among adolescents in ASEAN. Therefore, the aim of this study was to examine the relationship between self-reported leisure time physical inactivity frequency and sedentary behaviour and lifestyle correlates among school children in ASEAN.

## 2. Methodology

### 2.1. Description of Survey and Study Population

This study involved secondary analysis of existing cross-sectional school survey data from the GSHS from seven ASEAN (Cambodia, Indonesia, Malaysia, Myanmar, Philippines, Thailand, and Vietnam); the year of data collection for the different study countries differed, ranging from 2007 to 2013. The GSHS is a large school-based survey conducted primarily among students aged 13–17 years in developing countries, and its purpose is to provide data on health behaviours and protective factors among students to inform youth health programmes and policies [34]. All ASEAN from which GSHS datasets were publicly available were included in the analysis. Details and data of the GSHS can be accessed online [34]. A two-stage cluster sample design was used to collect data to represent all students in grades 6, 7, 8, 9, and 10 in each country [34]. At the first stage of sampling, schools were selected with probability proportional to their reported enrolment size [34]. In the second stage, classes in the selected schools were randomly selected and all students in selected classes were eligible to participate irrespective of their actual ages [34]. Students self-completed the questionnaires to record their responses to each question on a computer scannable answer sheet [34]. Consistent with the GSHS study protocol, in each participating country, the GSHS was approved by appropriate national government agencies and informed consent was obtained as appropriate from the students, parents and/or school officials [34].

### 2.2. Measures

The study variables used were from the GSHS [34] are described in Table 1. Body weight and height were recorded by self-report. The international age- and gender-specific child Body Mass Index (BMI), calculated as weight/height^2^ (kg/m^2^), cut-points were used to define overweight and obesity [35]. Adolescents were categorized as overweight if their BMI was > +1SD from the median for BMI for age and sex, and obese if their BMI was > +2SD from the median for BMI for age and sex [35]. Inadequate fruit consumption was defined as less than two or more servings a day and inadequate vegetable consumption is less than three or more servings a day [36]. The Patient-Centred Assessment and Counselling for Exercise Plus Nutrition (PACE+) self-report physical activity measure (used in this study) has been found to have acceptable validity for assessing non-achievement of the adolescent physical activity recommendations the measure, intraclass correlation 0.77 and correlation with accelerometer data, *r* = 0.40, *p* < 0.001 [37]. Physical inactivity was defined as obtaining less than 60 min of moderate to vigorous-intensity physical activity per day on at least 5 days per week [4,8]. “Sedentary” behaviour was defined as spending 3 or more hours per day sitting when not in school or doing homework [2,4]. The GSHS questionnaire was found to have good validity in a previous validation study: “Average agreement between test and retest was 77%, and average Cohen’s kappa was 0.47.” [38].

### 2.3. Data Analysis

Data analysis was conducted using STATA software version 13.0 (Stata Corporation, College Station, TX, USA). This software provides robust standard errors that account for the sampling design, *i.e*., cluster sampling owing to the sampling of school classes. In order to compare study samples across countries each country sample was restricted to the age group 13 to 15 years, younger and older participants were excluded from the analyses. Associations between socio-demographic indicators, health risk status and behaviour, poor mental health and protective factors among school children were assessed with odds ratios (OR). Logistic regression was used for the assessment of the impact of explanatory variables for physical inactivity and sedentary behaviour (binary dependent variables) for boys and girls separately. Independent variables found significant in relation to the outcome variables in bivariate analysis were included in the final multivariate model. In the analysis, weighted percentages are reported, and the sample that was asked the target question reflects the actual sample size. Both the reported 95% confidence intervals and the *p*-value are adjusted for the multi-stage stratified cluster sample design of the study. The *p*-value of less than 5% is used to indicate statistical significance.

**Table 1:** Variable description.

Variable	Question	Response Options
*Physical activity*	Leisure time physical activity was assessed by asking participants: “Physical activity is any activity that increases your heart rate and makes you get out of breath some of the time. Physical activity can be done in sports, playing with friends, or walking to school. Some examples of physical activity are running, fast walking, biking, dancing, football. Do not include your physical education or gym class.” “During the past 7 days, on how many days were you physically active for a total of at least 60 min per day?”	0 = 0 days to 8 = 7 days
*Sedentary behaviour*	Leisure time sedentary behaviour was assessed by asking participants about the time they spend mostly sitting when not in school or doing homework: “How much time do you spend during a typical or usual day sitting and watching television, playing computer games, talking with friends, or playing cards?”	1 = Less than 1 h *per* day… 3 = 3 to 4 h *per* day… 6 = 8 or more hours a day
*Physical education*	“During this school year, on how many days did you go to physical education (PE) class each week?”	1 = 0 days to 6 = 5 or more days
*Hunger (as proxy for socioeconomic status)*	“During the past 30 days, how often did you go hungry because there was not enough food in your home?”	1 = never to 5 = always
Health risk status and behaviour		
*Height*	“How tall are you without your shoes on?”	
*Weight*	“How much do you weigh without your shoes on?”	
*Fruits*	“During the past 30 days, how many times per day did you usually eat fruit?”	1 = I did not eat fruit during the past 30 days to 7 = 5 or more times *per* day
*Vegetables*	“During the past 30 days, how many times per day did you usually eat vegetables?”	I did not eat vegetables during the past 30 days to 7 0 = 5 or more times *per* day
*Bullied*	“During the past 30 days, on how many days were you bullied?”	1 = 0 days to 7 = All 30 days
*Current smoking cigarettes*	“During the past 30 days, on how many days did you smoke cigarettes?”	1 = 0 days to 7 = All 30 days
*Current other tobacco use*	“During the past 30 days, on how many days did you use any other form of tobacco, such as chewing tobacco leaves?”	1 = 0 days to 7 = all 30 days
*Current alcohol use*	“During the past 30 days, on how many days did you have at least one drink containing alcohol?”	1 = 0 days to 7 = All 30 days
Mental health		
*Close friends*	“How many close friends do you have?”	1 = 0 to 4 = 3 or more
*Lonely*	“During the past 12 months, how often have you felt lonely?”	1 = never to 5 = always
*Suicidal ideation*	“During the past 12 months, did you ever seriously consider attempting suicide?”	1 = yes, 2 = no
Protective factors		
*School attendance (never miss school)*	“During the past 30 days, on how many days did you miss classes or school without permission?”	1 = 0 days to 10 or more days
*Peer support in school*	“During the past 30 days, how often were most of the students in your school kind and helpful?”	1 = never to 5 = always
*Parental or guardian supervision*	“During the past 30 days, how often did your parents or guardians check to see if your homework was done?”	1 = never to 5 = always
*Parental or guardian connectedness*	“During the past 30 days, how often did your parents or guardians understand your problems and worries?”	1 = never to 5 = always
*Parental or guardian bonding*	“During the past 30 days, how often did your parents or guardians really know what you were doing with your free time?”	1 = never to 5 = always

## 3. Results

### 3.1. Sample Characteristics

The total sample included 30,284 school children aged 13 to 15 years from seven ASEAN countries. The sample size in individual countries ranged from 1734 in Cambodia to 16,095 in Malaysia, 14,750 (48.5%) were boys and 15,430 (51.5%) were girls (see Table 2).

### 3.2. Prevalence of Physical Inactivity and Sedentary Behaviour

Overall, the prevalence of physical inactivity was 80.4% and sedentary behaviour 33.0%. There was variation in the prevalence of physical inactivity and sedentary behaviour among school children of the study countries, ranging in terms of physical inactivity from 74.8% in Myanmar to 90.7% in Cambodia, and in relation to sedentary behaviour from 10.5% in Cambodia and Myanmar to 42.7% in Malaysia. Overall, the prevalence of physical inactivity and sedentary behaviour was higher in girls than in boys. Exploring gender differences by study country, in four countries (Malaysia, Myanmar, Thailand, and Vietnam) the prevalence of physical inactivity was higher in girls than in boys, while there were no significant gender differences in Cambodia, Indonesia, and Philippines. Regarding country gender differences in terms of sedentary behaviour, the prevalence of sedentary behaviour was significantly higher in girls than boys in the Philippines, while this was the reverse in Myanmar (see Table 3). Table 4 describes the sample characteristics by independent variables and the prevalence of physical inactivity and sedentary behaviour (see Table 4).

### 3.3. Associations with Physical Inactivity

Multivariate logistic regression analysis, among both boys and girls found that not walking or biking to school, not attending physical education classes, inadequate vegetable consumption and lack of protective factors (peer and parental or guardian support) were associated with physical inactivity. In addition, in boys, lower socioeconomic status (sometime, mostly or always feeling hungry), coming from a low income or lower middle income country were positively and sedentary behaviour and loneliness negatively associated with physical inactivity (see Table 5).

### 3.4. Associations with Sedentary Behaviour

Multivariate logistic regression analysis, among both boys and girls found that older age (14 and 15 years old), coming from an upper middle income country, being overweight or obese, attending physical education classes, alcohol use, loneliness, peer support and lack of parental or guardian supervision were associated with sedentary behaviour. In girls, higher socioeconomic status (not sometime, mostly or always feeling hungry), not walking or biking to school, and being bullied was additionally associated with sedentary behaviour (see Table 6).

**Table 2:** Details of participating country samples included in the analyses (age 13–15 years only) (*N* = 30,284).

Study Country	Study Year	Country Income Level [39]	Gross National Income *per capita* [39] US$	Urban Population % [40]	Overall Response Rate %	Sample N (13–15 Years)	Male %	13 Years %	14 Years %	15 Years %	Secondary School Gross Enrolment Ratio [41] 2012/13
Cambodia	2013	LI	1020	21	85%	1734	49.1	24.0	38.1	37.9	NA
Indonesia	2007	LMI	3630	53	93%	2867	49.5	33.2	45.2	21.6	83%
Malaysia	2012	UMI	11,120	74	89%	16,095	49.5	33.3	33.6	33.0	71%
Myanmar	2007	LMI	NA	34	95%	1983	50.0	37.1	34.3	28.6	NA
Philippines	2011	LMI	3500	44	82%	3640	48.3	29.9	32.9	37.2	85%
Thailand	2008	UMI	5780	49	93%	2223	49.2	37.1	36.2	26.7	86%
Vietnam	2013	LMI	1890	33	96%	1742	46.6	1.0	47.8	51.2	NA

LI = Low income; LMI = Lower middle income; UMI = Upper middle income; NA = Not available.

**Table 3:** Prevalence and duration of physical activity and sedentary behaviour among school-going adolescents in ASEAN.

Study Country	Prevalence of Physical Activity			Prevalence of Sedentary Behaviour				Physical Inactivity (<5 Days/Weeks)			Sedentary Behaviour (≥3 h or More)		
	<3 Days	3–4 Days	5–7 Days	<1 h	1–2 h	3–4 h	≥5 h	All	Boys	Girls	All	Boys	Girls
	%	%	%	%	%	%	%	%	%	%	%	%	
Cambodia	82.4	8.3	9.3	63.7	25.8	6.1	4.4	90.7	89.4	92.0	10.5	11.0	10.0
Indonesia	60.3	15.3	24.4	23.0	43.3	21.6	12.1	75.6	74.0	77.1	33.7	33.0	34.2
Malaysia	59.9	18.4	21.7	23.9	33.3	24.7	18.0	78.3	71.7	84.8 ***	42.7	41.9	43.5
Myanmar	62.9	11.9	25.2	53.3	36.3	5.7	4.7	74.8	71.9	77.7 *	10.5	12.8 ***	8.2
Philippines	77.8	8.3	13.9	38.5	29.2	16.6	15.7	86.1	85.2	86.9	32.3	29.8	34.6 *
Thailand	59.2	16.7	24.1	27.6	32.8	21.6	18.0	75.9	66.7	84.8 ***	39.6	38.5	40.5
Vietnam	67.8	14.0	18.2	23.4	41.6	23.8	11.2	81.8	76.3	86.8 ***	35.0	33.8	36.0
All	67.2	13.2	19.6	31.2	35.8	19.5	13.5	80.4	76.5	84.1 ***	33.0	31.8	34.0 *

*** *p* < 0.001; * *p* < 0.05.

**Table 4:** Sample characteristics, physical inactivity and sedentary behaviour by independent variables.

Variable	Total Sample N (%)	Physical Inactivity		Sedentary Behaviour	
		Boys N (%)	Girls N (%)	Boys N (%)	Girls N (%)
Socio-demographics					
Age in years					
13	9130 (25.8)	3243 (78.3)	3851 (82.5)	1360 (26.6)	1448 (29.0)
14	10,972 (39.2)	3907 (77.0)	4667 (84.0)	1916 (33.3)	2112 (34.4)
15	10,182 (34.9)	3537 (74.7)	4351 (85.5)	1920 (34.1)	2124 (37.3)
Hunger					
Never	12,658 (43.1)	4230 (73.0)	5580 (83.4)	2039 (32.1)	2431 (34.9)
Rarely	7876 (25.3)	2825 (76.5)	3184 (84.2)	1548 (36.7)	1527 (37.1)
Sometimes/mostly/always	9663 (31.6)	3592 (81.0)	4079 (85.2)	1592 (28.1)	1716 (30.0)
Country income					
Upper middle income	18,318 (60.5)	6440 (68.7)	7433 (84.8)	3094 (39.9)	4018 (41.7)
Low income/Lower middle income	11,966 (39.5)	4247 (78.8)	5436 (83.9)	1302 (29.5)	1666 (31.8)
Health risk status and behaviour					
BMI Overweight or obese	4823 (9.9)	1871 (76.1)	1871 (82.4)	1060 (42.1)	977 (41.0)
Walk/bike to school in the past 7 days					
0	12,609 (37.0)	4706 (80.1)	5713 (88.0)	2134 (31.4)	2574 (36.9)
1–6	10,066 (32.1)	3568 (77.0)	4401 (84.1)	1613 (31.4)	1833 (31.6)
7	7472 (30.9)	2373 (71.8)	2714 (79.5)	1431 (32.5)	1264 (33.1)
Attendance of physical education ^1^					
0 days/week	2980 (12.3)	1377 (94.2)	1211 (94.4)	382 (16.0)	374 (21.3)
1 day/week	9277 (36.0)	3170 (80.3)	4233 (88.2)	1693 (35.6)	1990 (35.6)
2 or more days/week	10,779 (51.6)	3623 (75.6)	4493 (84.6)	2104 (34.5)	2279 (37.7)
Fruits (<2 servings)	17,450 (59.4)	6399 (78.8)	7566 (84.9)	2964 (30.3)	3231 (32.9)
Vegetables (<3 servings)	21,818 (73.0)	7821 (78.4)	9542 (85.0)	3668 (31.1)	4197 (34.5)
Bullied	7648 (35.6)	2966 (78.5)	3011 (84.8)	1427 (33.3)	1316 (36.3)
Current tobacco use	2661 (8.8)	1685 (78.1)	336 (86.7)	823 (34.9)	163 (40.9)
Current alcohol use	2337 (11.9)	1081 (76.2)	742 (85.3)	644 (42.4)	449 (50.7)
Poor mental health					
No close friends	957 (3.2)	426 (75.5)	355 (87.3)	194 (33.6)	152 (38.5)
Loneliness	2396 (9.7)	697 (72.2)	1163 (86.1)	425 (40.8)	633 (42.9)
Suicidal ideation	2325 (10.3)	685 (76.5)	1174 (84.8)	362 (33.5)	636 (41.8)
Protective factors					
School attendance (past 30 days)	21,164 (75.2)	6043 (74.5)	9193 (83.3)	3579 (31.3)	4265 (33.7)
Peer support in school (mostly/always)	12,024 (40.4)	3091 (68.1)	5636 (79.8)	1879 (35.1)	2918 (36.5)
Parental/guardian supervision (mostly or always)	7023 (31.3)	2323 (70.0)	2743 (78.4)	1026 (29.3)	947 (25.5)
Parental/guardian connectivity (mostly/always)	9960 (34.0)	3102 (71.4)	4096 (79.0)	1664 (31.6)	1799 (31.9)
Parental/guardian bonding (mostly/always)	12,668 (42.1)	3806 (70.4)	5515 (80.9)	2223 (33.4)	2628 (34.8)

^1^ Analysis for Cambodia, Malaysia, Philippines and Vietnam only (excluding Indonesia, Myanmar and Thailand).

**Table 5:** Associations between physical inactivity prevalence, health behaviour, mental health and protective factor variables in school going adolescents by gender from 7 ASEAN countries.

Variable	Boys		Girls	
	UOR (95% CI)	AOR (95% CI)	UOR (95% CI)	AOR (95% CI)
Socio-demographics				
Age in years				
13	1.00	1.00	1.00	
14	0.93 (0.77–1.12)	0.98 (0.82–1.18)	1.12 (0.89–1.40)	
15	0.82 (0.67–0.99) *	0.84 (0.68–1.03)	1.25 (0.97–1.61)	
Hunger				
Never	1.00	1.00	1.00	
Rarely	1.21 (1.03–1.41) *	1.10 (0.93–1.30)	1.06 (0.85–1.31)	
Sometimes/mostly/always	1.58 (1.34–1.86) ***	1.27 (1.05–1.54) *	1.15 (0.97–1.37)	
Country income				
Upper middle income	1.00	1.00	1.00	
Low income/Lower middle income	1.70 (1.45–1.98) ***	1.82 (1.54–2.15) ***	0.94 (0.76–1.16)	
Health risk status and behaviour				
BMI weight status				
Normal weight/Underweight	1.00		1.00	
Overweight or obese	0.99 (0.801.22)		0.86 (0.68–1.10)	
Walk/bike to school in the past 7 days				
0	1.00	1.00	1.00	1.00
1–6	0.83 (0.69–0.99) *	0.78 (0.65–0.95) *	0.72 (0.59–0.87) ***	0.71 (0.59–0.86) ***
7	0.63 (0.5–0.75) ***	0.65 (0.53–0.79) ***	0.53 (0.43–0.64) ***	0.55 (0.44–0.67) ***
Attendance of physical education ^1^				
0 days/week	1.00	1.00	1.00	1.00
1 day/week	0.25 (0.1–0.37) ***	0.32 (0.20–0.50) ***	0.44 (0.31–0.64) ***	0.54 (0.38–0.79) ***
2 or more days/week	0.19 (0.1–0.29) ***	0.28 (0.17–0.45) ***	0.33 (0.22–0.49) ***	0.48 (0.31–0.75) ***
Sitting (≥3 h/day)	0.67 (0.5–0.79) ***	0.70 (0.60–0.82) ***	0.87 (0.74–1.01)	
Fruits (<2 servings) (base = 2 or more servings)	1.38 (1.1–1.59) ***	1.16 (0.97–1.40)	1.16 (0.98–1.37)	
Vegetables (<3 servings) (base = 3 or more servings)	1.45 (1.2–1.68) ***	1.23 (1.01–1.49) *	1.29 (1.09–1.53) **	1.21 (1.02–1.43) *
Bullied	1.20 (1.0–1.43) *	1.03 (0.87–1.22)	1.09 (0.94–1.26)	
Current tobacco use (base = no)	1.12 (0.9–1.37)		1.23 (0.84–1.83)	
Current alcohol use (base = no)	0.97 (0.7–1.21)		1.11 (0.75–1.62)	
Poor mental health				
No close friends (base = yes)	0.95 (0.6–1.35)		1.31(0.85–2.01)	
Loneliness (base = no)	0.78 (0.6–0.99) *	0.69 (0.52–0.92) **	1.19 (0.89–1.59)	
Suicidal ideation (base = no)	1.10 (0.8–1.42)		1.38 (1.06–1.80) *	1.20 (0.90–1.59)
Protective factors				
School attendance (past 30 days)	0.71 (0.6–0.84) ***	0.84 (0.71–1.00)	0.80 (0.64–0.99) *	0.92 (0.74–1.64)
Peer support in school (mostly/always)	0.51 (0.4–0.58) ***	0.63 (0.53–0.76) ***	0.58 (0.49–0.69) ***	0.70 (0.59–0.82) ***
Parental/guardian supervision (mostly or always)	0.62 (0.5–0.72) ***	0.82 (0.7–0.96) *	0.58 (0.49–0.68) ***	0.75 (0.64–0.89) ***
Parental/guardian connectivity (mostly/always)	0.68 (0.6–0.77) ***	0.90 0.7–1.05)	0.59 (0.50–0.70) ***	0.75 (0.63–0.90) **
Parental/guardian bonding (mostly/always)	0.60 (0.5–0.69) ***	0.76 (0.6–0.89) ***	0.67 (0.56–0.81) ***	0.94 (0.78–1.13)

^1^ Analysis for Cambodia, Malaysia, Philippines and Vietnam only (excluding Indonesia, Myanmar and Thailand) *** *p* < 0.001; ** *p* < 0.01; * *p* < 0.05; UOR = Unadjusted Odds Ratio; AOR = Adjusted Odds Ratio; CI = Confidence Interval.

**Table 6:** Associations between sedentary behaviour prevalence, health behaviour, mental health and protective factor variables in school going adolescents by gender from 7 ASEAN countries.

Variable	Boys		Girls	
	UOR (95% CI)	AOR (95% CI)	UOR (95% CI)	AOR (95% CI)
Socio-demographics				
Age in years				
13	1.00	1.00	1.00	1.00
14	1.38 (1.16–1.65) ***	1.46 (1.21–1.77) ***	1.28 (1.04–1.59) *	1.29 (1.00–1.66) *
15	1.43 (1.15–1.77) ***	1.47 (1.16–1.86) ***	1.45 (1.16–1.81) ***	1.54 (1.20–1.96) ***
Hunger				
Never	1.00	1.00	1.00	1.00
Rarely	1.23 (1.05–1.44) *	1.16 (0.96–1.39)	1.10 (0.96–1.25)	0.98 (0.85–1.14)
Sometimes/mostly/always	0.83 (0.71–0.96) *	0.84 (0.69–1.02)	0.80 (0.68–0.93) **	0.79 (0.67–0.94) **
Country income				
Upper middle income	1.00	1.00	1.00	1.00
Low income/Lower middle income	0.66 (0.58–0.75) ***	0.63 (0.51–0.78) ***	0.65 (0.53–0.80) ***	0.68 (0.53–0.88) **
Health risk status and behaviour				
BMI weight status				
Normal weight/Underweight	1.00	1.00	1.00	1.00
Overweight or obese	1.63 (1.36–1.96) ***	1.57 (1.26–1.96) ***	1.38 (1.14–1.67) ***	1.28 (1.04–1.58) *
Walk/bike to school in the past 7 days				
0	1.00		1.00	1.00
1–6	1.00 (0.84–1.18)		0.79 (0.68–0.91) ***	0.82 (0.70–0.96) *
7	1.05 (0.89–1.24)		0.85 (0.70–1.02)	0.97 (0.78–1.20)
Attendance of physical education ^1^			
0 days/week	1.00	1.00	1.00	1.00
1 day/week	2.90 (1.99–4.23) ***	2.99 (1.95–4.59) ***	2.05 (1.55–2.70) ***	1.68 (1.26–2.24) ***
2 or more days/week	2.76 (1.92–3.98) ***	2.97 (2.01–4.40) ***	2.24 (1.58–3.17) ***	1.92 (1.33–2.78) ***
Fruits (<2 servings) (base = 2 or more servings)	0.83 (0.72–0.96) *	0.88 (0.75–1.03)	0.88 (0.80–0.98) *	0.98 (0.89–1.10)
Vegetables (<3 servings) (base = 3 or more servings)	0.89 (0.78–1.01)		1.08 (0.94–1.25)	
Bullied	1.09 (0.96–1.25)		1.15 (1.01–1.31) *	1.19 (1.03–1.37) *
Current tobacco use (base = no)	1.19 (0.99–1.41)		1.36 (1.02–1.81) *	1.00 (0.71–1.38)
Current alcohol use (base = no)	1.73 (1.45–2.07) ***	1.55 (1.24–1.93) ***	2.15 (1.78–2.60) ***	1.85 (1.49–2.30) ***
Poor mental health				
No close friends (base = yes)	1.09 (0.78–1.53)		1.22 (0.88–1.70)	
Loneliness (base = no)	1.53 (1.27–1.85) ***	1.41 (1.11–1.80) **	1.53 (1.30–1.80) ***	1.45 (1.21–1.74) ***
Suicidal ideation (base = no)	1.10 (0.87–1.39)		1.47 (1.21–1.78)	
Protective factors				
School attendance (past 30 days)	0.84 (0.74–0.95) **	0.90 (0.79–1.03)	0.81 (0.69–0.96) *	0.98 (0.77–1.24)
Peer support in school (mostly/always)	1.21 (1.05–1.39) **	1.26 (1.07–1.48) **	1.15 (1.01–1.30) *	1.32 (1.15–1.51) ***
Parental/guardian supervision (mostly or always)	0.81 (0.70–0.93) **	0.77 (0.66–0.89) ***	0.54 (0.46–0.63) ***	0.49 (0.40–0.60) ***
Parental/guardian connectivity (mostly/always)	0.94 (0.83–1.07)		0.82 (0.72–0.94) **	1.02 (0.89–1.18)
Parental/guardian bonding (mostly/always)	1.07 (0.94–1.22)		1.01 (0.91–1.14)	

^1^ Analysis for Cambodia, Malaysia, Philippines and Vietnam only (excluding Indonesia, Myanmar and Thailand); *** *p* < 0.001; ** *p* < 0.01; * *p* < 0.05; UOR = Unadjusted Odds Ratio; AOR = Adjusted Odds Ratio; CI = Confidence Interval.

## 4. Discussion

The current investigation explores the prevalence of leisure time physical inactivity and sedentary behaviour and their relationship to sociodemographic indicators, health risk status and behaviour, poor mental health and protective factors among school going adolescents from seven ASEAN member states. Overall, a high prevalence of physical inactivity (80.4%, 76.5% in boys and 84.1% in girls) and sedentary behaviour (33.0%) were found, which compares with previous investigations in mainly low and middle income countries [4,5,6]. However, this study found, among adolescents a higher prevalence of physical inactivity in Malaysia (78.3%), compared with a previous national study (57.3%) [13] and three local studies (20.8%–45%) among adolescents in Malaysia [18,42,43]. This is a great concern of very high physical inactivity calling for physical activity interventions among adolescent school children in ASEAN.

This study found cross-national variations in the prevalence of leisure time physical inactivity and sedentary behaviour. The prevalence of physical inactivity was the highest (>85%) in Cambodia and the Philippines and the lowest (75%–76%) in Indonesia, Myanmar and Thailand. The study was conducted in the study countries at different years, ranging from 2007 to 2013. This fact could have influenced some of the country differences. The prevalence of physical inactivity and sedentary behaviour was significantly higher (*p* < 0.001; analysis not shown) in countries where the study was conducted more recently (2011–2013: Cambodia, Malaysia, Philippines and Vietnam) than in countries where the study had been conducted earlier (2007–2008: Indonesia, Myanmar and Thailand). However, in the Philippines the GSHS was conducted in 2003, 2007 and 2011, and the prevalence of physical inactivity was 89.6%, 92.8% and 86.7%, respectively, and sedentary behaviour 31.6%, 32.7% and 33.9%, respectively, showing no significant differences over the years [44]. Among boys the study found that coming from a low income or lower middle income country, including Cambodia and the Philippines, increased the odds of physical inactivity, which is in agreement with a previous study among university students in 23 countries [45]. It is possible that adolescent boys from upper middle income countries had higher physical activity levels than students from low or lower middle income countries because of better access to sports or physical activity facilities [21]. The prevalence of sedentary behaviour was the highest (≥40%) in Malaysia and Thailand and the lowest (≤10.5%) in Cambodia and Myanmar. Compared with students from low and lower middle income countries, male and female students from upper middle income countries (Malaysia and Thailand) had significantly higher odds of sedentary behaviour. Possible other reasons for such country differences could be differences in urbanization rate, with the assumption that students from urban areas having a lower participation in physical activity (including walking or biking to school) than students from rural areas [5]. Yet, in the study countries with a low urbanization rate of less than 35% only Myanmar but not Cambodia and Vietnam had a low prevalence physical inactivity. However, in two study countries (Malaysia and Thailand) with a high urbanization rate (≥49%) also had a high prevalence of sedentary behaviour (≥40%). Moreover, in a study comparing physical activity patterns in rural with urban areas among adolescents in Vietnam, no significant urban-rural differences were found [46].

Further, overall the study found that the prevalence of leisure time physical inactivity and sedentary behaviour was higher in girls than in boys, which is largely in agreement with previous studies [6,13,16,17], and emphasises the importance of targeting physical inactivity and sedentary behaviour in female school-going adolescents. Increasing age was associated with increased odds to engage in sedentary behaviour, but not physical inactivity. This finding is confirmed in a longitudinal study among Vietnamese adolescents, with the largest increase of non-school sedentary behaviour being recreational screen time [47]. Unlike in previous studies [16,18,19], this study did not find an association between higher socioeconomic status (measured as never hungry) and physical inactivity, while among boys lower socioeconomic status was associated with physical inactivity. Among girls and among boys in bivariate analysis the odds for sedentary behaviour increased with higher socioeconomic status (never hungry), which is confirmed in a systematic review of school-aged children in African countries [16].

Regarding health risk status or excess body mass index (BMI), this study did not find an association between overweight or obesity and physical inactivity, which is confirming previous mixed results [13,17,20]. However, overweight or obesity was among both boys and girls associated with sedentary behaviour, which also confirms general studies showing that sedentary behaviours were positively associated with having a higher BMI [3,20,21]. In terms of health risk behaviour, not walking or biking to school, not attending physical education classes and inadequate vegetable consumption was associated with physical inactivity. These results were also found in some previous studies [17,24,25,26], and can be further utilized to promote physical activity among this population. Regarding sedentary behaviour, associations with substance use, being bullied among girls and not walking or biking to school among girls and attending physical education classes were found. The latter finding (restricted to four study countries: Cambodia, Malyasia, Philippines and Vietnam) supports the importance of providing good quality physical education at least once a week to promote physical activity in school adolescents. The association between substance use and sedentary behaviour has been confirmed in a number studies [5,23,27,28]. The positive association between attending physical education classes and sedentary behaviour has also been found in another study [25], which needs further research. Some studies [22,23] have tried to show that a group of negative health indices/health risk behaviour is associated with physical inactivity and sedentary behaviour.

A number of previous studies [29,30,31,32] have found an association between negative mental health indicators and physical inactivity and sedentary behaviour among adolescents. This finding has been confirmed for at least one poor mental health indicator, namely loneliness and sedentary behaviour, and suicidal ideation and physical inactivity among girls in bivariate analyses. Generally, there has been evidence of a protective association between physical activity and depression [32,48]. In terms of protective factors, this study found indicators of the lack of peer support and lack of parent of guardian support to be associated with physical inactivity, and lack of parent of guardian support to be associated with sedentary behaviour. Peer support was associated with sedentary behaviour. This may be explained by the possibility that sedentary activities like TV viewing and computer use are part of the peer culture [23]. In a previous review, Van Der Horst *et al.*, [33] also found positive associations between family and peer support and physical activity. This confirms the importance of familial factors in developing physical activity programmes.

### Limitations of the Study

This study had several limitations. Firstly, the GSHS only includes adolescents who are in school, which is not representative of all adolescents in a country. There may be differences in the occurrence of physical inactivity and sedentary behaviour between school-going and non-school going adolescents. As the questionnaire was self-completed, it is possible that some study participants biased their responses. It is possible that respondents, for example, overreported physical activity, as found in other studies among adolescents [49]. It should also be acknowledged that results should not be compared with similar data generated using objective measures, *i.e*., accelerometry. The questionnaire used in this study measured several concepts like poor mental health variables with single items, which are limited in their use as quantitative indices. Several other factors such as environmental factors [17] can be related to physical activity and were not assessed in this study, and should be assessed in future studies. In addition, the measure of experiencing hunger as a proxy for subjective socioeconomic status has its limitation. Furthermore, this study was based on data collected in a cross sectional survey and no causal conclusions can be drawn. The data are not sufficient to support conclusions about differences between countries or make any claims about the nature of the association between the behaviour and factors.

## 5. Conclusions

The study indicates that the prevalence of leisure physical inactivity and sedentary behaviour among school going adolescents in ASEAN countries is very high. Several socio-demographic indicators, health risk status and behaviour, poor mental health and protective factors were identified which may help guide physical activity promotion programmes in school-going adolescents in ASEAN.
